# Boron symbiotaxis: a new concept in host–microbiome communication

**DOI:** 10.3389/fmicb.2025.1719918

**Published:** 2026-01-06

**Authors:** Andreea Silvia Pîrvu, Andrei Biţă, Ion Romulus Scorei, Dan Ionuţ Gheonea, George Dan Mogoşanu

**Affiliations:** 1 Department of Biochemistry, Faculty of Medicine, University of Medicine and Pharmacy of Craiova, Craiova, Romania; 2 Drug Research Center, Faculty of Pharmacy, University of Medicine and Pharmacy of Craiova, Craiova, Romania; 3 Department of Pharmacognosy and Phytotherapy, Faculty of Pharmacy, University of Medicine and Pharmacy of Craiova, Craiova, Romania; 4 Department of Biochemistry, BioBoron Research Institute, S.C. Natural Research S.R.L., Podari, Romania; 5 Department of Gastroenterology, Research Center of Gastroenterology and Hepatology, University of Medicine and Pharmacy of Craiova, Craiova, Romania

**Keywords:** boron symbiotaxis, quorum sensing, autoinducer-2–borate, microbiota-accessible boron complexes, host–microbiome communication

## Abstract

Boron (B) participates in biological systems through reversible complexation with diols and phosphate esters, enabling it to stabilize labile furanosyl intermediates and to modulate the chemical landscape in which QS signals operate. Dietary B appears to enter two functional pools: plasma-accessible boron (PAB), composed of freely diffusible B(OH)_3_/B(OH)_4_^−^, and microbiota-accessible boron complexes (MABCs), formed *in situ* with polyols, chlorogenic acids, and fructans/inulins. MABCs persist at the mucus–epithelium interface, creating local reservoirs that can influence the persistence, diffusion, and recognition of AI-2-related molecules. Here we propose a structured, testable framework—“boron symbiotaxis”—to describe how B may stabilize 4,5-dihydroxypentane-2,3-dione (DPD)-derived intermediates (and, in defined lineages, form the furanosyl borate diester AI-2B), localize chemical potential via MABCs at the mucosal surface, and orient microbial behavior toward particular community states. This framework does not assume that enhanced AI-2 signaling is inherently beneficial; QS coherence can also support opportunistic growth or virulence, depending on ecological context. We therefore outline experimental approaches—including speciation-resolved ^11^B–NMR, targeted LC–MS for AI-2/AI-2B, and bacterial reporter strains with defined AI-2 receptors—to discriminate beneficial from adverse outcomes. Altogether, we highlight B as a chemically plausible modulator of QS architectures in the gut, propose falsifiable predictions linking diet → B speciation → AI-2 dynamics → host phenotypes, and identify scenarios in which B-driven stabilization or localization could be either advantageous or detrimental.

## 1 Introduction

The symbiotic relationship between humans and their gut microbiome is orchestrated through an intricate web of chemical exchanges (Bassler, 1999; Chigozie et al., 2025). Central to this dialogue are quorum sensing (QS) molecules—autoinducers that coordinate microbial density, cooperation, and interspecies behaviors. Among them, the autoinducer-2 (AI-2) system has been described as a broadly conserved “*lingua franca*” of bacteria. In several lineages and environmental contexts, the biologically active species is a borate-complexed furanosyl signal (AI-2B), whose stability and receptor-competent conformation depend on boron (B) availability (Chen et al., 2002).

Unlike classical cofactors such as Mg^2+^, Zn^2+^, or Fe^2+/3+^, B operates primarily through dynamic covalency, forming reversible complexes with *cis*-diols (in sugars, polyols, polyphenols) and interacting cooperatively with phosphate esters. This chemistry stabilizes labile, information-bearing molecules, biases equilibria between furanosyl and open-chain forms, and, in the case of AI-2B, incorporates B directly into the architecture of a QS signal. We refer to B’s directional role in this network as “boron symbiotaxis”: the stabilization of key signals, the localization of chemical potential via microbiota-accessible boron complexes (MABCs), and the orientation of host–microbe dialogue toward cooperative, resilient states (Biţă et al., 2022).

B bioavailability to the gut ecosystem follows two complementary routes. Plasma-accessible boron (PAB), largely present as freely diffusible boric acid (BA), is absorbed in the upper intestine and distributed systemically. In parallel, MABCs form in the intestinal lumen from dietary polyols, polyphenols, and chlorogenic acids (CGAs), generating poorly absorbed borate–diol conjugates that persist at the mucosal interface (Biţă et al., 2022, Biţă et al., 2023a,b). In this mini-review, we use the term “speciation” in its chemical sense, to denote the distribution of B among molecular forms (e.g., B(OH)_3_, B(OH)_4_^−^, borate–diol complexes). Local chemical speciation, rather than total intake, is therefore poised to influence when and where QS signals operate at the host–microbiome boundary.

Several broader reviews have discussed B nutrition, B-containing natural products, and B–microbiota interactions, including work from our own group and others. In contrast, the present mini-review has a narrower, mechanistic scope: we focus on how B chemistry may intersect with AI-2 signaling architectures and how PAB *versus* MABCs might shape QS behavior at the mucosal interface. We introduce “boron symbiotaxis” as a hypothesis that integrates these observations into testable predictions, rather than as a new physiological law.

## 2 Boron chemistry in biological contexts—foundation for symbiotaxis

B shows distinctive behavior in biological systems through the formation of reversible borate esters with hydroxyl-rich substrates and context-dependent interactions with phosphate esters. In aqueous solution, B is present mainly as B(OH)_3_ (trigonal, sp^2^) and, at higher pH, as B(OH)_4_^−^ (tetrahedral, sp^3^). Interconversion between these forms underlies B ability to complex vicinal diols and stabilize furanosyl intermediates (Sivaev and Bregadze, 2014; Hunter et al., 2019; Nori et al., 2022). Because borate esters form and dissociate rapidly at physiological ionic strength, B can stabilize ribose and related furanoses against degradation (Ricardo et al., 2004; Scorei and Cimpoiaşu, 2006; Scorei, 2012), extend the lifetimes of phosphate esters in the presence of adjacent diols (Kim et al., 2016), and facilitate condensation reactions under wet–dry or freeze–thaw cycles (Franco et al., 2019; Franco and da Silva, 2021).

Complex formation is strongly influenced by geometry and pH. B forms five- and six-membered cyclic esters with 1,2-/1,3-diols; multidentate polyols and catechols generally show higher apparent affinities than many aldohexoses, and ketoses often bind more strongly than aldoses (Rietjens and Steenbergen, 2005). Functionally, B does not interact equally with all carbohydrates: it tends to favor furanosyl conformations and stabilize chemically fragile sugar intermediates. These trends help explain why diet-derived polyols and polyphenols efficiently bind B in the intestinal lumen and contribute to MABCs formation (Table 1).

**Table 1:** Qualitative affinity of boron for different diol classes and biological relevance.

Diol class	Representative ligands	Relative affinity	Factors that increase affinity	Typical complexes	Biological relevance (PAB *versus* MABCs)
Monosaccharides (aldo-pentoses/hexoses)	Ribose, arabinose, glucose	Low–moderate; affinity varies widely by ring form; open-chain aldohexoses bind weakly	*cis*-1,2/−1,3-diol geometry; furanose forms; higher pH (≥7)	Ribose–borate esters; furanosyl stabilization	Contributes to MABCs formation; biases ribose/furanose
Monosaccharides (keto-sugars)	Fructose, tagatose	Moderate–high; often stronger binding than aldohexoses; relevant to nutritional complexes	Multiple *cis*-diols; ketose geometry; neutral–alkaline pH	Fructoborates (e.g., calcium fructoborate)	Stable MABCs species in gut lumen; dietary origin
Polyols (sugar alcohols)	Mannitol, sorbitol, xylitol	High; strong competitors for boron; shape MABCs speciation via diet	Multiple adjacent *cis*-diols; neutral–alkaline pH; high local concentration	Mannitol–borate, sorbitol–borate chelates	Robust MABCs reservoirs; slow exchange, local persistence
Polyphenols (catechols)	Catechol; caffeic acid; chlorogenic acids	High–very high; can outcompete sugars; major driver of B localization in lumen	*Ortho*-diol (*ortho*-dihydroxy) motif; deprotonation at pH ~ 7–8	Chlorogenoborates; catechol–borate	Potent MABCs formers; stabilize signals, localize B
Glycosides/polysaccharides with *cis*-diols	Pectins (RG-II motif), hemicelluloses	Moderate–high (multivalent); biological exemplar of borate “rivets”; mechanistic analogy for stabilization	Multivalency; structured binding pockets; Ca^2+^ coordination	Borate crosslinks in plant RG-II	Structural analogies; inform B as crosslinker concept
Phosphate esters (with neighboring diols)	Sugar–phosphates, nucleotide–sugars	Context-dependent; cooperative effects reduce hydrolysis; reversible “protective group” behavior	Proximity of *cis*-diol; microenvironment; pH	Mixed borate–diol–phosphate arrangements	Potential protection of phosphate esters; supports signaling persistence

B, Boron; MABCs, Microbiota-accessible boron complexes; PAB, Plasma-accessible boron; RG-II, Rhamnogalacturonan-II.

Although B is not a classical phosphate ligand, neighboring diols permit cooperative interactions that decrease the hydrolytic lability of phosphate mono- and diesters. Mixed borate–diol–phosphate adducts and associated hydrogen-bond networks can limit water access to phosphorus centers, increasing the activation barrier for spontaneous cleavage (Aydoğmuş et al., 2014; Hirakawa et al., 2022). Because borate esters are reversible, this protective effect is conditional and sensitive to pH, ionic strength, ligand geometry, and divalent cations (Mg^2+^, Ca^2+^) (Van Duin et al., 1985).

Several biological systems illustrate that this chemistry is compatible with living matrices. In plants, borate–diol crosslinks in polysaccharides such as rhamnogalacturonan II contribute to wall mechanics and integrity (González-Fontes et al., 2008; Brini and Landi, 2022). In microorganisms, a furanosyl borate diester (AI-2B) is recognized by LuxP in some *Vibrio* spp., indicating that borate complexation can be integral to QS signal identity (Chen et al., 2002; Zhang et al., 2025). B-containing drugs (e.g., bortezomib, tavaborole) further show that reversible B chemistry can be harnessed in controlled biological contexts (Das et al., 2022; Soriano-Ursúa et al., 2024).

Within this context, PAB consists of small, diffusible species absorbed in the proximal intestine and distributed systemically, whereas MABCs arise locally when diet-derived ligands form poorly absorbed conjugates that persist at the mucosal surface and release B by reversible exchange (Biţă et al., 2024, 2025). The balance between PAB and MABCs shaped by dietary ligands, luminal pH and ionic milieu, transit time, and mucosal adsorption—likely contributes to QS-relevant speciation and to the structure and resilience of microbial communities.

## 3 AI-2 and the borate-complexed signal (AI-2B)—a model for symbiotaxis

AI-2 is generated by LuxS-mediated conversion of S-ribosylhomocysteine to 4,5-dihydroxypentane-2,3-dione (DPD), a reactive intermediate that spontaneously rearranges in aqueous solution into a pool of interconverting furanosyl isomers. AI-2 thus denotes an ensemble of DPD-derived species rather than a single molecule. DPD itself can be viewed both as a QS intermediate and as an inevitable product of the activated methyl cycle (Holmes et al., 2009).

Under favorable conditions, one subset of this ensemble forms a furanosyl borate diester (AI-2B). In marine *Vibrio* spp. that express LuxP-type receptors and inhabit borate-rich seawater, this B-containing form (often described as (2*S*,4*S*)-2-methyl-2,3,3,4-tetrahydroxytetrahydrofuran (*S*-THMF)–borate) is the best characterized signal: B contributes to stabilization of the ligand conformation and to high-affinity receptor recognition. By contrast, in many enteric bacteria and other lineages, receptors such as LsrB and more recently identified dCache_1_- and gammaproteobacterial periplasmic sensor (GAPES1)-domain proteins appear to respond predominantly to unboronated AI-2 isomers (Frando et al., 2025; Liu et al., 2025a,b).

Recent large-scale genomic and metatranscriptomic analyses of the human gut microbiome (Fan et al., 2025) support this view: LuxS is widely distributed, but most gut-associated lineages encode receptors that are, to current knowledge, specific for unboronated AI-2 (LsrB, dCache_1_, GAPES1), whereas B-dependent LuxP systems are essentially absent from typical intestinal consortia. These data reinforce the distinction between a chemically broad AI-2 ensemble and the more restricted, context-dependent AI-2B species.

Importantly, Fan et al. (2025) also report that unhealthy states are associated with a shift toward *Enterobacteriaceae* expressing AI-2 receptors, indicating that high AI-2 signaling can accompany dysbiosis rather than symbiosis. In the framework developed here, such findings are consistent with a scenario in which unboronated AI-2 dominates in low-B or dysbiotic contexts, whereas AI-2B formation would require adequate luminal B availability and appropriate speciation.

Thus, while the chemical requirement for B in AI-2B formation is well established in specific LuxP/seawater contexts, the prevalence and quantitative contribution of AI-2B, relative to unboronated AI-2, across taxa and niches remain incompletely defined and are likely to be context-dependent rather than universal. In the gut lumen, where B levels, pH, and ligand composition differ markedly from seawater, unboronated AI-2 forms are expected to predominate, with AI-2B representing one possible, but not obligatory, state within the broader DPD-derived ensemble. Even in systems where receptors do not require B, B may still influence signaling indirectly by stabilizing selected DPD-derived species and modestly extending their lifetime and effective diffusion distance.

## 4 Microbiota-accessible boron complexes: a two-compartment framework

Not all ingested B is functionally equivalent. PAB refers to freely diffusible B(OH)_3_/B(OH)_4_^−^ absorbed in the upper intestine and distributed via the circulation (Hunter et al., 2019). In contrast, MABCs are luminal, reversible borate–diol conjugates formed *in situ* with dietary ligands such as polyols, CGAs and other polyphenols, and fructans/inulins (Biţă et al., 2023a, 2024, 2025). Because these complexes are poorly absorbed, they tend to concentrate at the mucus–epithelium interface. This partitioning into a systemic, diffuse pool (PAB) and a local, luminal pool (MABCs) helps determine where QS-relevant chemistry occurs, largely independent of total elemental intake (Table 1).

Within the lumen, BA forms several classes of reversible esters. Chlorogenoborates derived from CGAs in coffee and fruits are stable at physiological pH but remain sensitive to pH, ionic strength, and enzymatic activity (Biţă et al., 2023a). Fructoborates and other polyol–borates (e.g., sorbitol- or mannitol-based complexes, fructan/inulin adducts) bind B via multiple *cis*-diols, lowering dissociation rates and favoring retention (Hunter et al., 2019). Engineered nutraceuticals such as calcium fructoborate (CaFB) add further motifs expected to behave as MABCs reservoirs (Biţă et al., 2022). These species can interact with O-glycan-rich mucus, which presents clustered *cis*-diols, creating regions where B is held near the epithelium and released gradually by reversible exchange.

By increasing the local lifetime and concentration of intermediates that participate in AI-2/AI-2B signaling, MABCs are expected to favor taxa that respond to these cues (e.g., LuxP-type lineages) and thereby act as ecological filters that bias communities toward states compatible with an intact epithelial barrier. At the same time, although complexation chemistry is well documented, direct *in vivo* evidence that MABC-derived B specifically drives AI-2B formation and QS coherence is still lacking. Addressing this gap will require speciation-aware analytical strategies, including ^11^B–nuclear magnetic resonance (NMR) to resolve coordination environments and targeted liquid chromatography–mass spectrometry (LC–MS) for AI-2/AI-2B and DPD isomers in promptly stabilized samples.

## 5 Conceptual framework: boron symbiotaxis

Several strands of evidence—the reversible chemistry of B with diols and phosphate esters, the exemplar of AI-2B in LuxP systems, and the local generation of MABCs in the gut—support the idea that B can influence when and where QS signals persist. For clarity, these processes can be grouped into three interrelated functions: (i) stabilize—B helps stabilize chemically labile intermediates through reversible borate esters or mixed borate–phosphate assemblies (when AI-2B forms, B becomes an integral part of the signal itself); (ii) localize—B is concentrated through MABCs that accumulate at the mucus–epithelium interface and release B gradually, in contrast to the more diffuse distribution of PAB; and (iii) orient—the combination of increased persistence and localized availability can modulate microbial interactions, influencing biofilm architecture, resource sharing, and colonization behavior, effects that the host perceives through changes in barrier function and immune tone.

We do not propose “boron symbiotaxis” as a new physiological program, but as a testable set of mechanistic predictions grounded in established B chemistry and known QS architectures. The extent of B’s effects is expected to depend on local pH, buffer capacity, ionic strength, Ca^2+^/Mg^2+^, availability of ligands (polyols, CGAs, other polyphenols), and ecological–physiological variables such as mucus turnover, transit, receptor distribution, and inflammation or permeability. Chemical speciation, rather than total B intake, should therefore correlate most closely with QS coherence.

**Figure 1:**
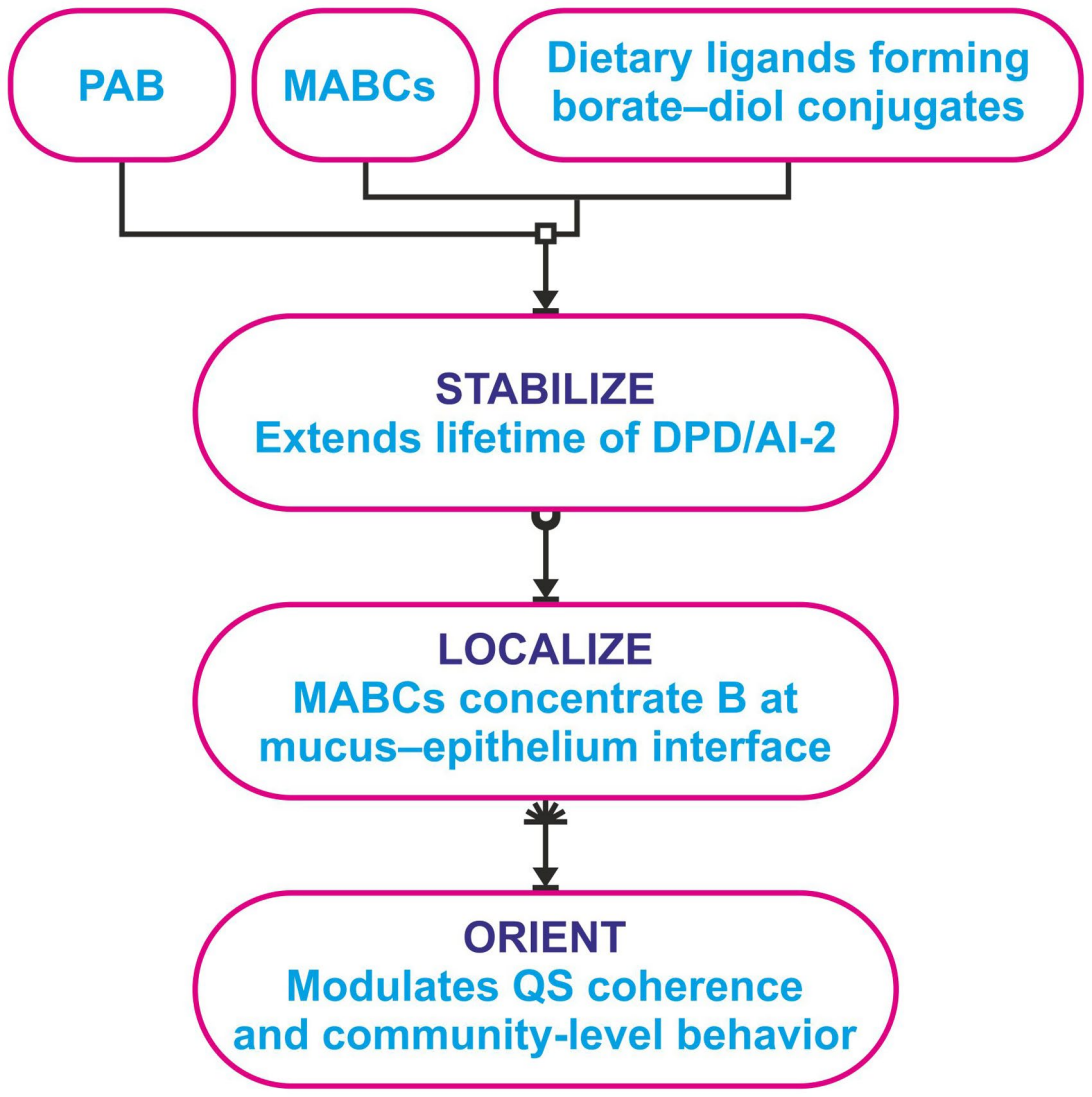
Overview of PAB (freely diffusible B(OH)_3_/B(OH)_4_^−^) and MABCs (luminal borate–diol complexes formed with dietary ligands) as distinct contributors to B speciation at the mucosal interface. The stabilize–localize–orient triad summarizes three mechanistic hypotheses: (i) Stabilization of selected DPD-derived intermediates, (ii) localization of B through MABC retention in mucus, and (iii) orientation of QS-relevant interactions. This schematic illustrates how diet-driven B speciation may influence AI-2/AI-2B dynamics.

Dietary contexts that favor MABC formation should enhance local AI-2/AI-2B signaling relative to PAB-dominated conditions at equivalent elemental doses (Biţă et al., 2025). Receptor-based reporter assays are expected to track luminal speciation, and a practical MABCs/PAB index may help compare interventions. Host outcomes linked to barrier integrity and immune tone are likewise predicted to vary with these chemical parameters. A schematic summary of the stabilize–localize–orient framework and its relationship to PAB and MABCs is provided in Figure 1.

## 6 Discussion

The contribution of B to AI-2 signaling is likely to be conditional rather than universal. In some lineages that express LuxP-type receptors and inhabit neutral to mildly alkaline niches environments, a furanosyl borate diester (AI-2B) appears necessary for optimal recognition. In many other bacteria, however, receptors respond to non-boronated AI-2 isomers. Mapping this diversity will require receptor-based reporter assays across taxa, combined with environmental metadata (pH, buffer capacity, ionic strength, divalent cations) that determine whether borate ester formation stabilizes a recognition-competent geometry or remains incidental.

Importantly, enhanced AI-2 activity should not be equated with beneficial or cooperative outcomes. Recent work on AI-2 signaling shows that expression of LuxS and AI-2-responsive receptors does not consistently correlate with improved host status and may, in some settings, accompany dysbiosis or virulence (Fan et al., 2025; Liu et al., 2025b; Zhang et al., 2025). We therefore treat AI-2 as a communication channel whose functional outcome depends on context, not as an inherently “symbiotic” signal. Within this framing, B is positioned as a modifier of signal chemistry rather than a determinant of whether QS promotes or undermines host–microbiome balance.

Functional QS assays are essential to bridge chemistry and biology. Reporter strains that differentially sense boronated *versus* non-boronated AI-2—such as LuxP/LuxQ-based *Vibrio harveyi* systems, LsrB-dependent enteric bacteria (*Escherichia coli*, *Salmonella*), and dCache_1_- or GAPES1-domain constructs in *Pseudomonas*—can be exposed to controlled gradients of B speciation, from simple BA to defined MABCs. Quantifying AI-2/AI-2B distributions by LC–MS and ^11^B–NMR under these conditions will allow direct tests of whether dietary-like B environments generate measurable differences in QS output.

Diet and gut ecology provide a tractable axis of variation. Diets rich in polyols and polyphenols (e.g., CGAs from coffee and fruits, fructans/inulins, sugar alcohols) are expected to favor MABCs-dominated environments, whereas highly-processed patterns may favor a more PAB-dominated state. Transit time and short-chain fatty acid production, which lower luminal pH, further modulate speciation along the tract. These gradients can serve as “experiments of opportunity” when combined with standardized chemical analytics and receptor-based assays.

Potential risks and adverse scenarios deserve explicit consideration. Although the stabilize–localize–orient triad may enhance communication fidelity in many commensal communities, increased QS coherence is not universally protective. Several pathogens—including *Pseudomonas*, *Vibrio*, *Klebsiella*, and certain *Enterobacteriaceae*—use AI-2 or AI-2-like cues to coordinate virulence, biofilm maturation, or antimicrobial tolerance. In such contexts, prolonged lifetime or enhanced clarity of AI-2 intermediates could, in principle, sustain or amplify undesirable behaviors. Controlled assays should therefore include commensal and opportunistic strains, matched gradients of B speciation, readouts for virulence and biofilm structure, and defined countermeasures such as pH modulation, ligand dilution, or transient inhibition of QS pathways.

For translational work, pragmatic intervention studies could compare MABCs-rich formulations (e.g., CaFB or chlorogenoborate-containing complexes) with BA at matched elemental B. Such trials would combine chemical endpoints (^11^B–NMR speciation, AI-2/AI-2B by LC–MS) with biological endpoints (Lux-based reporter assays, barrier-related markers such as zonulin, and inflammatory or metabolic panels), alongside diet records and safety monitoring.

In summary, the symbiotaxis framework proposes that boron can influence QS dynamics by stabilizing selected intermediates (and, in some lineages, by forming part of the AI-2B signal molecule), by shaping local speciation via MABCs at the mucus–epithelium interface, and by affecting microbial community states linked to barrier integrity and immune tone. These ideas are advanced not as established mechanisms, but as a coherent set of hypotheses. Demonstrating a reproducible chain from diet, B speciation, to QS readouts and host phenotypes will be essential to determine when and to what extent B can be used to modulate microbiome resilience and human health, and to confirm or refute the proposed concept of “boron symbiotaxis.”

## Data Availability

The original contributions presented in the study are included in the article/supplementary material, further inquiries can be directed to the corresponding author.
